# Revealing 3-hydroxybenzoate-4-hydroxylase from *Pseudarthrobacter phenanthrenivorans* Sphe3 via In-Cell NMR analysis and molecular cloning

**DOI:** 10.1007/s10123-026-00856-7

**Published:** 2026-06-26

**Authors:** Stamatia Asimakoula, Alexandra Primikyri, Epameinondas Tsagogiannis, Anna-Irini Koukkou

**Affiliations:** 1 Laboratory of Biochemistry, Sector of Organic Chemistry and Biochemistry, Department of Chemistry, University of Ioannina, Ioannina, 45110 Greece; 2 Laboratory of Organic Chemistry, Sector of Organic Chemistry and Biochemistry, Department of Chemistry, University of Ioannina, Ioannina, 45110 Greece

**Keywords:** *Pseudarthrobacter phenanthrenivorans* Sphe3, Flavoprotein monooxygenases, 3-hydroxybenzoate 4-hydroxylase, Protocatechuic acid, In-cell NMR, Actinobacteria

## Abstract

This study aims at the functional and kinetic characterization of 3-Hydroxybenzoate-4-Hydroxylase (3HB4H) from *Pseudarthrobacter phenanthrenivorans* Sphe3. Bioinformatic sequence and structural analyses suggested that Asphe3_36590 gene encodes a putative 3HB4H, a finding supported by transcriptional data of a ~ 2000-fold induction when Sphe3 cells were grown on 3-Hydroxybenzoate (3-HBA) compared to glucose. The recombinant 3HB4H is highly specific for 3-HBA and follows Michaelis–Menten kinetics, with Km and Vmax values of 72.6 µM and 42.5 µM × min^− 1^, respectively, at pH 8.5 and 25 ◦C. It accepts both NADH and NADPH as cofactors and primarily hydroxylates 3-HBA to protocatechuate (PCA), while also converting 4-Hydroxybenzoate and gentisic acid. Combining heterologous expression with in-cell NMR using living *Escherichia coli* (*E. coli)* cells overexpressing 3HB4H, demonstrated that 3HB4H encoded by Asphe3_36590 gene catalyzes the hydroxylation of 3-HBA. Real-time NMR revealed rapid intracellular conversion of 3-HBA to PCA, providing direct functional validation of the enzyme in a living cellular environment. Sphe3 3HB4H represents the first biochemically characterized single-subunit FAD-depended hydroxylase (monooxygenase) in Actinobacteria. Furthermore, these findings highlight the exceptional power of in-cell NMR to capture enzymatic activity non-invasively, allowing direct observation of substrate consumption and PCA formation without the need for derivatization or chromatographic separation. This approach offers a powerful platform for investigating enzymatic activity and metabolic transformations in living cells.

**Graphical Abstract:**

**Figure:**
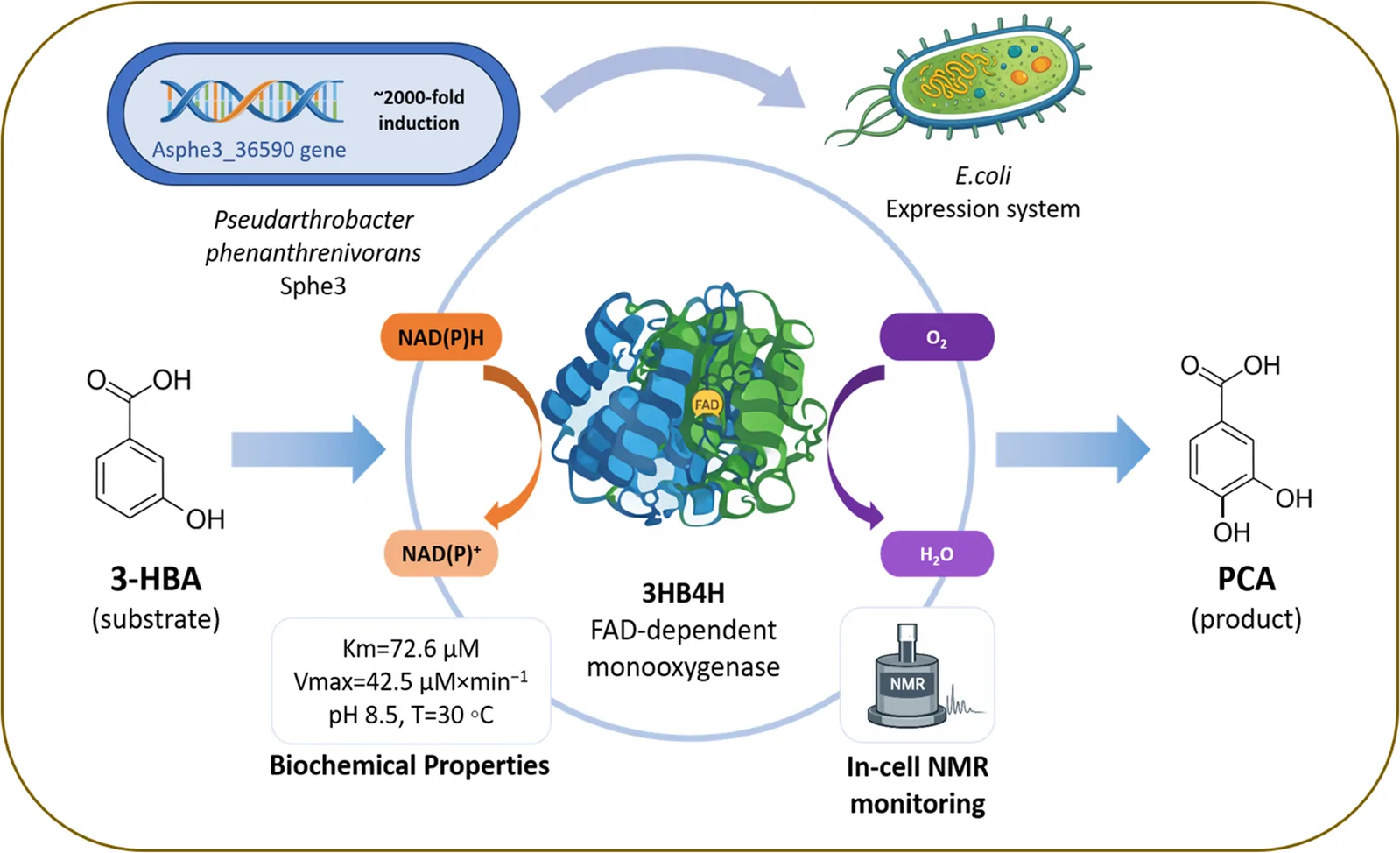

**Supplementary Information:**

The online version contains supplementary material available at https://doi.org/10.1007/s10123-026-00856-7.

## Introduction

*Pseudarthrobacter phenanthrenivorans* Sphe3, a Gram-positive soil bacterium formerly isolated from a creosote polluted area, has proven to be a rather catabolically versatile bacterial strain as it is able to grow in the presence of various aromatic compounds, as reported in our previous studies (Kallimanis et al. 2007; Vandera et al. 2015; Tsagogiannis et al. 2021).

Apart from genes encoding for various dioxygenases (Vandera et al. 2012; Tsagogiannis et al. 2021; Asimakoula et al. 2022), the Sphe3 genome harbors several genes encoding for FAD-depended monooxygenases predicted to participate in the catabolism of phenolic compounds, such as phenol and hydroxybenzoic acids. Among these, the Asphe3_36590 gene, annotated as a phenol 2-monooxygenase, has been previously studied for its possible involvement in phenol hydroxylation (Asimakoula et al. 2023). However, the enzymatic function of its gene product remains uncharacterized.

Bioinformatic analysis revealed that the predicted protein product of Asphe3_36590 gene from Sphe3 shares high sequence identity with putative phenol hydroxylases (monooxygenases) from other Arthrobacter strains (Asimakoula et al. 2023), however none of these enzymes has been characterized. Moreover, when compared with FAD-depended hydroxylases whose structures have been studied, the predicted protein product of Asphe3_36590 gene from Sphe3 shares higher sequence identity at the amino acid level with the 3-hydroxybenzoic acid hydroxylases.

3-hydroxybenzoic acid (3-HBA, also known as m-hydroxybenzoate) is a very common environmental compound as it is an intermediate in the catabolism of lignin, which in turn could act as a sustainable feedstock for the manufacture of biofuels, bioplastics and other commodity chemicals (Paone et al. 2020; Erickson et al. 2022). 3-HBA is also a common intermediate in the biodegradation of very widespread groups of environmental pollutants, such as various PAHs, m-cresol, alkylphenols and other phenolic compounds (Gao et al. 2005; Park et al. 2007; Sucharitakul et al. 2013; Ren et al. 2014; Yao et al. 2023).

3-HBA can either be hydroxylated in a *para*- position, by a 3-Hydroxybenzoate-6-Hydroxylase (3HB6H) to form gentisate (Chen et al. 2018), or in an *ortho*-position, by a 3-Hydroxybenzoate-4-Hydroxylase (3HB4H) to form protocatechuate (Chang and Zylstra 2008). It seems that the hydroxylation of 3-HBA to gentisate is a rather widespread pathway among bacterial strains as various 3HB6H enzymes have been studied, including the ones from the strains *Burkholderia cepacian* (Wang et al. 1987), *Corynebacterium glutamicum* (Yang et al. 2010), *Rhodococcus jostii* (Montersino and Van Berkel 2012), *Polaromonas naphthalenivorans* (Park et al. 2007), *Klebsiella pneumoniae* (Suarez et al. 1995), *Pseudomonas alcaligenes* (Gao et al. 2005), *Micrococcus* sp. (Rajasekharan et al. 1990), as well as the 3HB6H from the halophilic *Martelella* strain AD-3 (Chen et al. 2018). On the other hand, the hydroxylation of 3-HBA to protocatechuate appears to be preferred notably less frequently by bacterial strains, as only two bacterial 3HB4Hs have been characterized to date, from the strains *Comamonas testosteroni* KH122 and *Chromohalobacter* sp. HS-2 (Michalover and Ribbons 1973; Hiromoto et al. 2006; Kim et al. 2012).

In-cell NMR spectroscopy offers the unique advantage of studying biochemical processes as well as molecular interactions within the native dynamic cellular system. This methodology mitigates discrepancies between in vitro and in vivo behaviors, thereby bridging the gap between conventional in vitro experiments and physiological conditions (Luchinat and Banci 2023). Its versatility extends to various biological systems, including prokaryotic and mammalian cells, making it broadly applicable (Primikyri et al. 2018; Teng et al. 2024). Unlike traditional approaches, which often rely on isolated proteins or metabolites, in-cell NMR allows for real-time monitoring of molecular dynamics, structural changes, and enzymatic activities within living cells. This provides direct insights into the behavior of biomolecules in their natural biological context, including effects from cellular crowding, endogenous post-translational modifications, and complex biochemical networks (Luchinat and Banci 2022; Theillet and Luchinat, 2022).

In-cell NMR offers a unique window for understanding biochemical reactions and the behavior of proteins, nucleic acids, metabolites and small molecules inside living organisms with an unparalleled resolution (Gerez et al. 2022; Zhao et al. 2023; Víšková et al. 2024). With its ability to preserve cell viability and enable non-invasive, time-resolved analyses of metabolic processes and drug interactions, in-cell NMR represents a powerful approach in cellular and molecular biology. One of the most promising applications of this methodology is the ability to monitor enzymatic reactions in real-time within living *E. coli* cells (Ma et al. 2014; Razew et al. 2024).

Hereby we demonstrate the functional and kinetic characterization of a FAD-depended oxidoreductase encoded by the Asphe3_36590 gene from *P. phenanthrenivorans* Sphe3 using traditional biochemical approaches complemented by the innovative in-cell NMR methodology for the real-time monitoring of the enzymatic reaction within living *E. coli* BL21 (DE3) cells overexpressing the respective gene.

## Materials and methods

### Bacterial strains and growth conditions

*P*. *phenanthrenivorans* Sphe3 was grown in minimal medium M9 (MM M9) (Vandera et al. 2012) in the presence of 3-HBA (5mM) as the sole source of carbon and energy, on a rotary shaker agitated at 180 rpm, at 30 °C. *E. coli* cells, DH5a and BL21 (DE3), were cultured in lysogeny broth (LB) medium in the presence of Kanamycin, when necessary, for selection.

### Phylogenetic analysis and structure prediction of 3HB4H from strain Sphe3

#### 3D structure prediction of 3HB4H

Template search with BLAST and HHblits has been performed against the SWISS-MODEL template library (SMTL, last update: 2024-11-14, last included PDB release: 2024-11-08). During enzyme structure prediction, building a comprehensive and accurate Multiple Sequence Alignment (MSA) is the most critical step. Both BLAST (or its iterations like PSI-BLAST) and HHblits act as two entirely different lenses for looking at evolutionary history. By combining them, close, high-precision relatives and distant, structural cousins can be captured to map out an enzyme’s shape. BLAST populates the MSA with immediate family members to secure local alignment constraints, while HHblits brings in distant evolutionary relatives to deepen the alignment. Moreover, BLAST searches for an easy-to-use, high-identity structure. If BLAST comes up empty-handed (no close templates exist), HHblits takes over to execute fold-recognition and threading, finding a distantly related enzyme that shares the same catalytic domain architecture despite a completely different sequence (Remmert et al. 2011).

The target sequence was searched with BLAST against the primary amino acid sequence contained in the SMTL. A total of 42 templates were found. An initial HHblits profile has been built using the procedure outlined in Steinegger et al. 2019; followed by 1 iteration of HHblits against Uniclust30 (Mirdita et al. 2017). The obtained profile has then been searched against all profiles of the SMTL. A total of 5094 templates were found. For each identified template, the template’s quality has been predicted from features of the target-template alignment. The templates with the highest quality have then been selected for model building. Models are built based on the target-template alignment using the online version of ProMod3 as implemented in SWISS-MODEL (Studer et al. 2021). Coordinates which are conserved between the target and the template are copied from the template to the model. Insertions and deletions are remodeled using a fragment library. Side chains are then rebuilt. Finally, the geometry of the resulting model is regularized by using a force field. The global and per-residue model quality has been assessed using the QMEAN scoring function (Studer et al. 2020). The structural sanity and stereochemical quality of the final tertiary model were rigorously evaluated. Global and local geometric qualities were estimated using QMEAN scoring. To complement the statistical potentials, a detailed backbone conformation analysis was performed via Ramachandran plot calculations using the MolProbity pipeline within the SWISS-MODEL structure assessment server. The distribution of the dihedral torsion angles (ϕ and ψ) was mapped to identify any steric clashes or geometric outliers.

#### Phylogenetic analysis

Sequence alignments were performed using the BLAST program (Altschul 1997) on the NCBI website, and amino acid sequences that exhibited similarity with 3HB4H were retrieved from protein databases (GenBank and RCSB Protein Data Bank) and aligned using CLUSTALW (accessed on November 2024) (Thompson et al. 1994). To construct the phylogenetic tree and conduct molecular evolutionary analyses, MEGA v.7.0 was used applying the neighbor-joining method (Kumar et al. 2016).

### Preparation of Sphe3 cell extracts

Sphe3 cells were grown in 3-HBA (5 mM) and harvested at the mid-exponential phase of the growth curve by centrifugation at 6000 × g for 15 min at 4 °C, and were subsequently washed with 50 mM Tris-HCl buffer (pH 8) containing 1 mM dithiothreitol (DTT) and 10 mM phenylmethylsulfonyl fluoride (PMSF), resuspended in 2 mL of the same buffer and disrupted with a mini Bead Beater (Biospec Product, Bartlesville, OK, USA) (10 times of 1 min periods using zirconium beads 0.1 mm in diameter). The homogenate was centrifuged at 12,000 × g for 20 min at 4 °C and the collected supernatant was kept on ice to prevent the inactivation of the enzymes and was further used as the cell-free extract for the enzyme assays. Protein concentration in the crude enzyme was determined spectrophotometrically by applying the Bradford method using the Bio-Rad reagent (Bio- Rad Laboratories, Hercules, CA, USA) and bovine serum albumin (BSA) (Amresco Inc., Solon, OH, USA) as the standard (Bradford 1976).

### Quantitative real-time PCR (RT-qPCR)

Total RNA isolation was performed using the NucleoSpin^®^ RNA Isolation kit by MACHEREY-NAGEL (Düren, Germany) according to manufacturer’s instructions with slight modifications on the sample homogenization procedure. Specifically, Sphe3 cells were grown in MM M9 supplemented with 5 mM 3-HBA as the sole source of carbon and energy. The cell pellet was obtained by harvesting Sphe3 culture at the mid-exponential phase of growth and centrifuging at 6000 × g for 15 min at 4 °C. Then, the cell pellet was resuspended in 100 µL TE buffer (10 mM Tris-HCl, 1 mM EDTA; pH 8) containing 10 mg/mL lysozyme by vigorous vortexing. The resulting solution was incubated for 1 h at 37 °C.

cDNA synthesis was performed using the PrimeScript™ RT Reagent Kit with gDNA Eraser (Perfect Real Time, Takara Bio Inc., Kusatsu, Shiga, Japan) according to the manufacturer’s instructions and stored at -20 °C. cDNA was further diluted and used as a template at a final concentration of 2.5 ng. The expression levels of the target genes were quantified by RT-qPCR in the CFX Connect Real-Time PCR Detection System (Bio-Rad, United States) using the Kapa SYBR Fast qPCR Kit Master Mix (2×) Universal (Kapa Biosystems, Wilmington, MA, USA), performed as described previously (Vandera et al. 2012).

Primers used in the RT-qPCR of the present study are listed in Table S1. The efficiency (E) of one cycle RT-qPCR in the exponential phase was found to be 1.89–2.05 (E = 10(-1/slope) (Pfaffl 2001; Corbella and Puyet 2003) with correlation factors 0.9918 < R^2^ < 0.9949. The housekeeping gene *gyr*β was used as the reference gene and gene expression levels in glucose were used as a calibrator. The results were analyzed by the relative quantification method (Applied Biosystems, 1997).

### Cloning and heterologous expression of the Asphe3_36590 gene

Asphe3_36590 is a 1950 bp gene located onto Sphe3 chromosome that encodes the putative 3HB4H. Genomic DNA was isolated from Sphe3 cells grown on LB according to the standard JGI (California) protocol for bacterial genomic DNA isolation using cetyltrimethylammonium bromide (CTAB) (William et al. 2012). Asphe3_36590 gene was amplified by a polymerase chain reaction (PCR) in a thermal cycler (PTC-100, version 7.0 – MJ Research Inc., Waltham, MA, USA) programmed at 95°C for 5 min, 30 cycles of 98°C for 20 s, 64°C for 15 s, 72°C for 2 min, and a final elongation step of 72°C for 5 min, using KAPA HiFi (Roche Diagnostics, Basel, Switzerland) polymerase enzyme and specific primer pair (forward primer: 5’-AAGTGAGGATT*CATATG*CAGTTCC-3’, reverse primer: 5’-ACAGGAC*AAGCTT*GACCGTCAG-3’) carrying binding sites for NdeI and HindIII (underlined) restriction enzymes. The amplified DNA product was purified by NucleoSpin Extract 2 in 1 (Macherey-Nagel GmbH & Co. KG, Dueren, Germany), cloned into pBlueScript SK(+) vector (Invitrogen, Thermo Fischer Scientific, Watham, MA, USA) for nucleotide sequence verification and subsequently subcloned with NdeI/HindIII restriction in the overexpressing vector pET29c(+) (Novagene Co., Ltd., Beijing, China) resulting in the recombinant plasmid designated pET29c::36,590. The pET29c::36,590 was used to transform BL21 (DE3) cells for heterologous expression as described elsewhere (Vandera et al. 2012). Host transformation was carried out according to either Hanahan (for *E. coli* DH5a strain) (Hanahan 1983) or Chung and Miller (for *E. coli* BL21 (DE3) strain) (Chung and Miller 1988). Gene nucleotide sequence verification was carried out by VBC Genomics Bioscience Research GmbH (Wien, Germany). Restriction enzyme digestion, ligation reaction, and agarose, as well as SDS-PAGE electrophoresis were carried out using the standard methodology (Sambrook et al. 2003). Compute MW/pI tool of ExpASy server was used to predict the molecular weight of the protein. Densitometric analysis and molecular weight verification of the target protein were performed using the web-based version of ImageJ (ImageJ.JS/ImJoy) (Schneider et al. 2012; Ouyang et al. 2019). Briefly, the gel image was converted to 8-bit grayscale, and lane profiles were generated to determine the migration distances (in pixels) of both the molecular weight (*MW*) marker bands and the protein of interest. A standard curve was constructed by plotting the relative migration distance (x) against the logarithm (log_10_) of the known molecular weights of the ladder (Unstained Protein Standard, New England Biolabs). Linear regression analysis yielded the equation *y = -0.002x + 2.0407*, R^2^ = 0.9855, where y represents log_10_(*MW*). Based on this linear model, the experimental molecular weight of the target protein was calculated to be approximately 69 kDa.

### Crude cell extract preparation and 3HB4H purification

The abovementioned *E*. *coli* BL21(DE3)-pET29c(+) host-vector overexpression system was used for the overexpression of the Asphe3_36590, as described elsewhere (Vandera et al. 2012), with the modification of incubating the cells under vigorous agitation at 16 °C and harvesting the cells by centrifugation 16–18 h after the addition of IPTG. For the enzyme purification, cell free supernatant was applied to a 20 mL HisPrep FF 16/10 column (GE Healthcare, Chicago, IL, USA) using and AKTA FPLC system (GE Healthcare, Chicago, IL, USA), as described before (Tsagogiannis et al. 2021).

### Enzyme assays, kinetic measurements

The purified enzyme was incubated with various concentrations of FAD (0.01-1 mM) in order to achieve maximum specific enzymatic activity. The activities of 3HB4H, 4-hydroxybenzoate hydroxylase, salicylate 1-hydroxylase and phenol monooxygenase under standard conditions were estimated spectrophotometrically by monitoring the oxidation of NADH/NADPH at 340 nm, as described elsewhere (Durham et al. 1984; Kukor and Olsen 1992; Mulla et al. 2016; Lubbers et al. 2021; Westphal et al. 2021; Xu et al. 2021) with slight modifications, as follows: Activity was assayed in 1 ml of reaction mixture containing the reaction buffer (50 mM TrisHCl, pH 8), FAD (for the purified enzyme, 0.25 mM), NADPH/NADH (0.2 mM), substrate 3-ΗΒΑ (0.03 to 0.3 mM) and the enzyme solution. 4-ΗΒΑ, 2-ΗΒΑ, gentisic acid, phthalic acid, PCA and phenol were tested as alternative substrates at various concentrations. In each case, the reaction was initiated by the addition of the respective substrate. One unit of enzyme activity was defined as the amount required to catalyze the formation of 1 µmol of NADH to NAD^+^ per minute at room temperature.

The kinetic parameters K_m_ and V_max_ values were determined in a broad concentration range of 3-HBA substrate varying from 0.03 to 0.3 mM, with EnzFitter (Biosoft) software by nonlinear fitting of the Michaelis–Menten equation. All kinetic values are the means of three separate measurements.

The effect of pH in the activity of recombinant 3HB4H was investigated in the pH range of 4 to 12 at room temperature, as described elsewhere (Tsagogiannis et al. 2021). To determine the optimal temperature for the 3HB4H, the above assays were performed in a temperature range of 10 to 80 ^ο^C in the optimal pH. To study the effect of activation and inhibition by metal ions, enzyme was incubated with different concentrations of several divalent cations (Ag^+^, Ba^2+^, Ca^2+^, Co^2+^, Fe^2+^, Mg^2+^, Mn^2+^, Zn^2+^) as described before (Tsagogiannis et al. 2021).

All enzyme assays were performed at 25 ^ο^C using a Shimadzu UV-1201 spectrophotometer (Triad Scientific, Inc., Manasquan, NJ, USA).

### NMR analysis of 3HB4H-catalyzed reactions

All NMR experiments were performed on a Bruker NEO 500 MHz spectrometer (University of Ioannina, Greece) equipped with a cryoprobe (NEO-TCPI). Samples were prepared in 90% H₂O/10% D₂O and transferred to 5 mm NMR tubes. ^1^H NMR spectra were acquired at 310 K using 64 scans. Water suppression was achieved using the excitation sculpting pulse sequence with a 2 ms water-selective 180° sinc-shaped pulse and relaxation delay was set at 1s. NMR spectra were processed and analyzed using TopSpin 4.2 software (Bruker Biospin, Rheinstetten, Germany). To maintain oxygen availability during NMR monitoring, all experiments were performed using a tube cap containing a small opening to permit gas exchange with the external environment. All spectra within a given experimental series were plotted using the same scaling ratio unless otherwise indicated.

To investigate the conversion of 3-HBA catalyzed by 3HB4H under increasing levels of biological complexity, NMR experiments were performed using (i) purified recombinant 3HB4H, (ii) crude cell extract of *E.coli* cells overexpressing 3HB4H, and (iii) living recombinant *E. coli* cells overexpressing 3HB4H.

#### NMR analysis using purified recombinant 3HB4H

The enzymatic conversion of 3-HBA by purified recombinant 3HB4H was monitored by NMR spectroscopy. Reaction mixtures were prepared in 0.5 mL Tris-HCl buffer (pH 8.0) containing 0.3 mM 3-HBA, 0.02 µM purified 3HB4H, 0.2 mM NADH, and exogenous 0.25 mM FAD. NADH was introduced into the enzyme-substrate mixture as the primary reducing equivalent, facilitating electron transfer and exogenous FAD was added as a necessary flavin cofactor to ensure optimal enzymatic activity and functionality. Following the addition of 3-HBA, the reaction mixture was immediately transferred to the NMR spectrometer and sequential ^1^H NMR spectra were acquired from the same sample over a period of 2.5 h. Spectra were recorded at 5 min, 10 min, 15 min, 1.2 h, 1.5 h and 2.5 h after reaction initiation to monitor substrate consumption and product formation in situ. For the structural characterization of the reaction product, 2D NMR experiments were performed under the same conditions. 2D TOCSY experiments were acquired with a mixing time of 60 ms.

The progress of the enzymatic reaction was monitored by time-resolved ^1^H NMR spectroscopy acquired directly from the same reaction mixture. The evolution of the reaction was assessed using the relative integrals of well-resolved signals corresponding to the H4 proton of 3-HBA and the H5 proton of PCA within each spectrum. Changes in the product-to-substrate integral ratio were monitored from sequential spectra recorded over time.

#### NMR analysis using *E. coli* crude cell extracts

Crude cell extract was prepared from *E. coli* cells which overexpressed 3HB4H enzyme, as described above. NMR samples were prepared by adding 0.3 mM of 3-HBA to the cell extract and transferred to a 5 mm NMR tube. Exogenous FAD was not added to the solution of the crude extract, since the omission of protein extraction and purification steps preserved the native flavin cofactor within the extract. The enzymatic conversion of 3-HBA was monitored by sequential ^1^H NMR measurements under the conditions described in Sect. NMR analysis using purified recombinant 3HB4H.

#### In-cell NMR analysis using *E. coli* recombinant cells

5 ml of recombinant *E. coli* cell seeds were inoculated into 5 mL LB media in the presence of 25 µg/mL Kanamycin and grown with shaking at 37 °C for overnight. *E. coli* BL21-pET29c::36,590 cells were cultivated following IPTG induction of Asphe3_36590 overexpression. Cell cultures were centrifuged at 2500 RPM for 20 min at 4 °C. The cell pellets were resuspended in 1 mL buffer (M9 in 90%H_2_O/10%D_2_O, pH 7.0). The cell suspension was centrifuged again, and the process was repeated at least three times. The substrate 3-HBA was added into the cell’s suspension and 500 uL of final solution were placed in 5 mm NMR tubes for data acquisition. The final OD_600_ of the cell suspension was calculated at 2.1 by multiplying the measured OD_600_ with the fold of dilution. Sequential ^1^H NMR spectra were acquired from the same sample at defined time intervals to monitor substrate conversion in living cells. Control cells, lacking the recombinant pET29c::36,590 plasmid, were harvested after reaching an optical density (OD_600_) of 5.5 and 0.2 mM of 3-HBA were added to the living cells suspension.

To assess whether 3HB4H-mediated hydroxylation of 3-HBA occurred in the extracellular medium, a supernatant control experiment was performed. *E. coli* cells overexpressing 3HB4H were incubated with 0.2 mM 3-HBA under the same conditions used for the in-cell biotransformation assays. Following incubation, the cell suspension was centrifuged (2500 RPM for 20 min), and the supernatant was carefully separated from the cell pellet. A fresh aliquot of 3-HBA was then added to the recovered supernatant to a final concentration of 0.2 mM. The reaction mixture was monitored by ^1^H NMR spectroscopy at 5 and 45 min after substrate addition.

#### Cell viability assessment for in-cell NMR experiments

*E. coli* cell viability was assessed using colony-plating assay before and after the NMR experiments. The number of colonies grown on LA plates supplemented with Kanamycin and inoculated with cells prior to the in-cell NMR procedure was compared to the number of colonies formed from cells after the NMR experiment. A modification in the assay consists of dividing each plate into quadrants, onto which droplets of serially diluted inocula are spotted, allowing the resulting colonies to be countable.

### Results-discussion

### Phylogenetic analysis and structure prediction of 3HB4H from strain Sphe3

In order to identify the function of the Asphe3_36590 gene from *P. phenanthrenivorans* Sphe3 various bioinformatics software tools were applied. Initially, a BlastP alignment with characterized proteins was conducted to help infer the function of the protein based on the known function of its alignment partners. The predicted protein product of Asphe3_36590 gene from Sphe3 consists of 634 amino acids and shares 49–51% sequence identity at the amino acid level with the 3ΗΒ4Ηs from *Comamonas testosteroni* GZ39 and *Chromohalobacter* sp. HS-2, 21–25% sequence identity with 3HB6Hs, 22–28% sequence identity with salicylate hydroxylases, 27% sequence identity with phenol monooxygenases and 20–24% sequence identity with 4HB3H (Table S2).

The InterPro tool for Classification of protein families was also used and the analysis conducted for the Asphe3_36590 protein matched 2 entries for representative families and 6 aromatic-ring hydroxylase (flavoprotein monooxygenase) signatures and also, 5 entries for characteristic domains. The family entries included phenol 2-monooxygenase, also known as phenol hydroxylase (PHHY) and RIFMO-like-Rifampicin monooxygenase. Among the characteristic domains there are 2 FAD- and NAD(P)-binding domains and 2 distinct domains belonging to phenol hydroxylase family (Figure S1, Table S3). Asphe3_36590 is annotated as a putative phenol 2-monooxygenase (Gene ID: 650468243, JGI/IMG database), but both sequence alignments and cluster analysis support for a 3-hydroxybenzoate 4-hydroxylase.

Both aforementioned enzymes are classified into group A of flavoprotein monooxygenases (FMPOs). FMPOs are redox enzymes that utilize a flavin mononucleotide (FMN) or flavin adenine dinucleotide (FAD) cofactor to activate dioxygen (O_2_) and are categorized in 6 groups based on distinct structural and functional properties. Group A members are single-component enzymes, which use NAD(P)H as external electron donor and act mainly as aromatic hydroxylases involved in the microbial degradation of (poly)aromatic hydrocarbons and in natural products biosynthesis (Paul et al. 2021). The most extensively studied enzymes of group A flavoprotein monooxygenases are 4-hydroxybenzoate 3-hydroxylases (Westphal et al. 2021), while 3-hydroxybenzoate 4-hydroxylases are scarcely studied.

Interestingly, group A FMPOs enzymes, such as 3-hydroxybenzoate 6-hydroxylase (3HB6H) and 3-hydroxybenzoate 4-hydroxylase (3HB4H), can react with the same substrate and result in the formation of a different product (Hiromoto et al. 2006). Specifically, aerobic degradation of 3-HBA occurs via its hydroxylation in a *para*- position or in an *ortho*-position by either 3HB6H or 3HB4H to form gentisate and PCA, respectively, which are further funneled to the central metabolism of the cell (Chen et al. 2018).

**Fig. 1:**
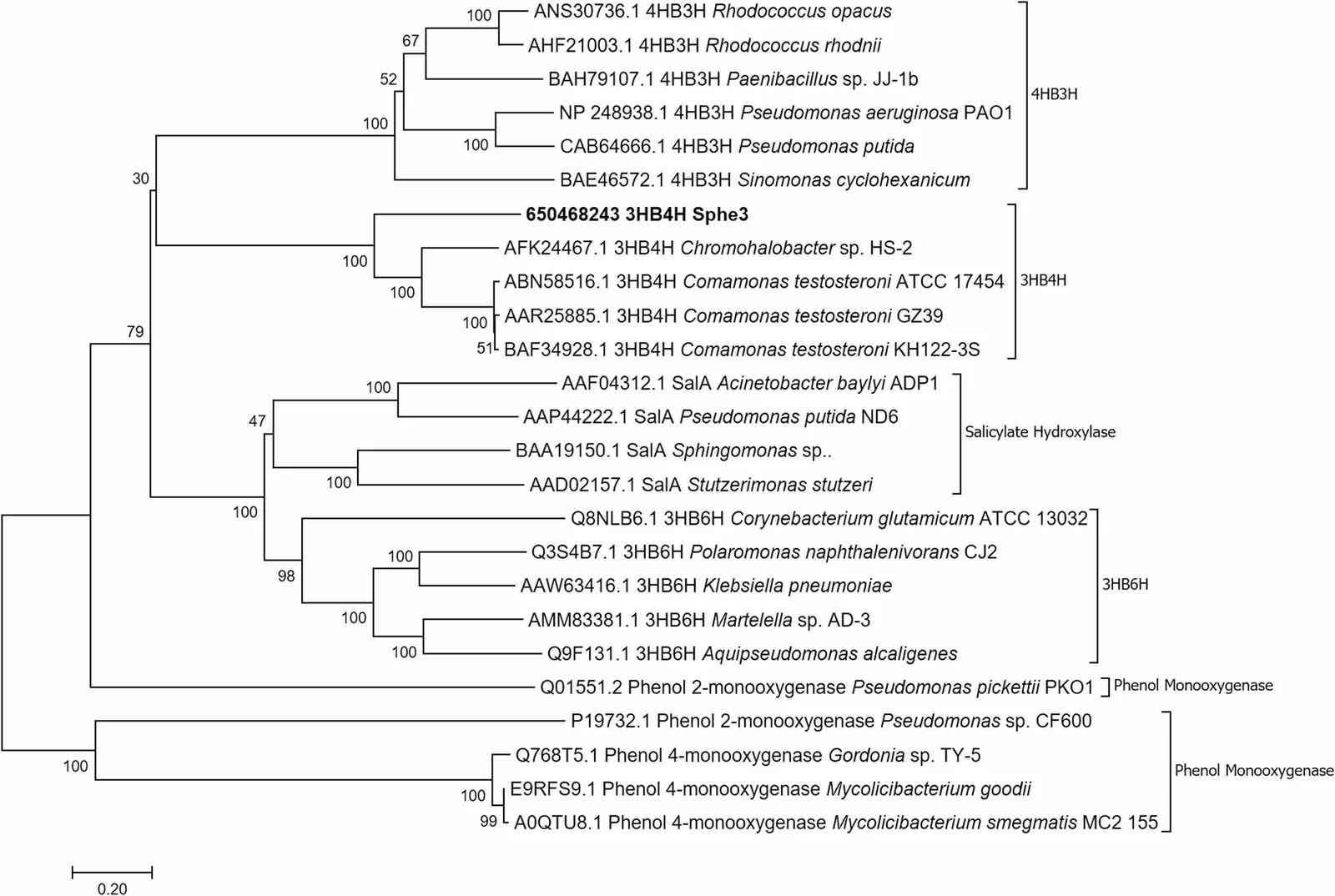
Evolutionary relationships of taxa. The evolutionary history was inferred using the Neighbor-Joining method (Saitou and Nei 1987). The optimal tree with the sum of branch length = 12.15691257 is shown. The percentage of replicate trees in which the associated taxa clustered together in the bootstrap test (500 replicates) are shown next to the branches (Felsenstein 1985). The tree is drawn to scale, with branch lengths in the same units as those of the evolutionary distances used to infer the phylogenetic tree. The evolutionary distances were computed using the Poisson correction method (Zuckerkandl and Pauling 1965) and are in the units of the number of amino acid substitutions per site. The analysis involved 25 amino acid sequences. All ambiguous positions were removed for each sequence pair. There was a total of 740 positions in the final dataset. Evolutionary analyses were conducted with MEGA7 (Kumar et al. 2016)

Other characteristic enzymes of group A FPMOs are 4-hydroxybenzoate-3-hydroxylases, salicylate 1-hydroxylases and phenol 2-monooxygenases (Michalover and Ribbons 1973; Hiromoto et al. 2006; Chang and Zylstra 2008; Montersino et al. 2011; Huijbers et al. 2014; Chen et al. 2018), therefore, a phylogenetic tree was constructed that included 3-hydroxybenzoate 4-hydroxylases, salicylate hydroxylases, 3-hydroxybenzoate 6-hydroxylases, 4-hydroxybenzoate 3-hydroxylases and phenol monooxygenases along the putative 3HB4H from Sphe3 (Fig. 1).

This phylogenetic analysis confirmed that the putative 3HB4H from Sphe3 is most closely related to the 3HB4Hs from *Comamonas testosteroni* and *Chromohalobacter* sp. HS-2. It is worth mentioning that the bacterial metabolism of 3-HBA through the formation of PCA appears to be relatively rare, as so far only these two 3HB4Hs have been studied (Michalover and Ribbons 1973; Kim et al. 2012).

**Fig. 2:**
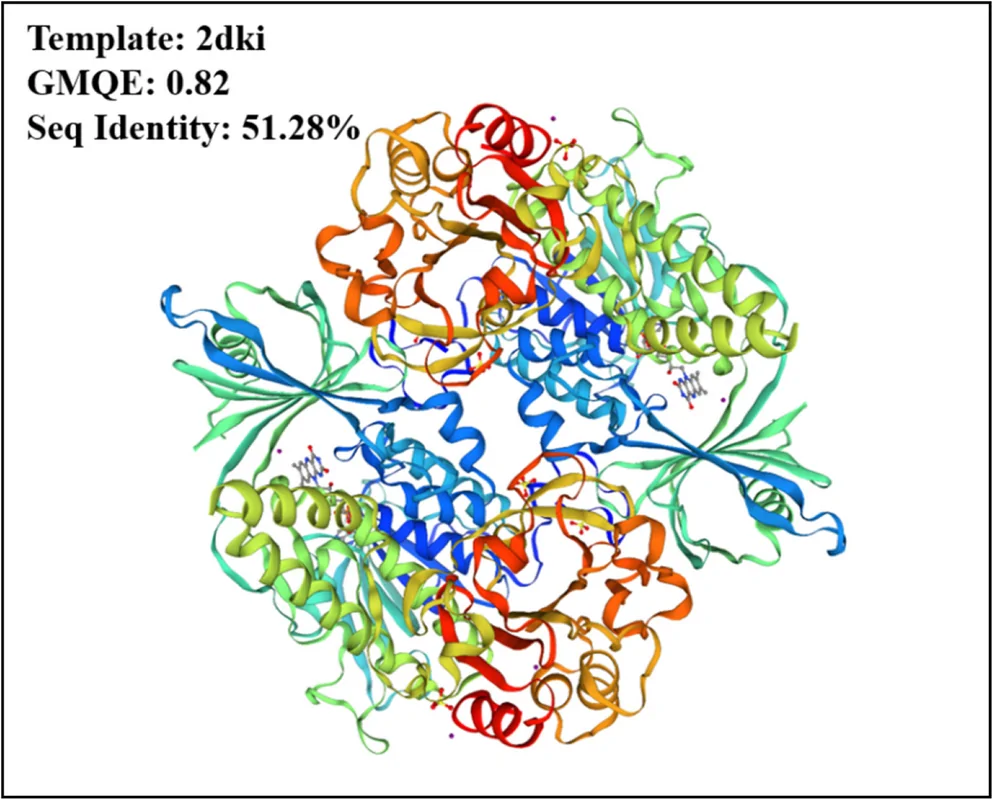
3d structure prediction of Asphe3_36590 peptide sequence, as derived from SWISS-MODEL analysis on the ExpASy platform, using the enzyme: 3-hydroxybenzoic acid 4-hydroxylase from *Comamonas testosteroni* (SMTL ID: 2dki.1). Top left text shows the enzyme code, the evaluation constant of the GMQE model (1 = optimal) and the similarity (%) of the sequence under investigation to that of the model (Image coloured by rainbow N → C terminus)

Moreover, the SWISS-MODEL template library (SMTL version 2024-11-14, PDB release 2024-11-08) was searched with BLAST (Camacho et al. 2009) and HHblits (Steinegger et al. 2019) for evolutionary related structures matching the target sequence in Table S2. The enzyme was compared with reported crystal structures of homologous proteins and showed 52% identity with 3-hydroxybenzoate 4-hydroxylase from *Comamonas testosteroni* and 38% identity with phenol 2-monooxygenase from *Trichosporon cutaneum* (SWISS-MODEL of ExpASy) and its structure was predicted using the enzyme 3-hydroxybenzoic acid 4-hydroxylase from *Comamonas testosteroni* (SMTL ID: 2dki.1) as template (Fig. 2). Validation of the predicted 3D structure demonstrated highly favorable stereochemical parameters. Ramachandran plot analysis revealed that 94.53**%** of all amino acid residues fall within the core favored regions, and 4.67**%** occupy the additionally allowed regions (Figure S2). Crucially, only 0.80**%** of the residues were flagged as stereochemical outliers. These metrics confirm that the backbone conformation of the modeled protein is sterically sound and provides a highly reliable structural foundation for downstream interpretations.

### Transcriptional analysis of Asphe3_36590

**Fig. 3:**
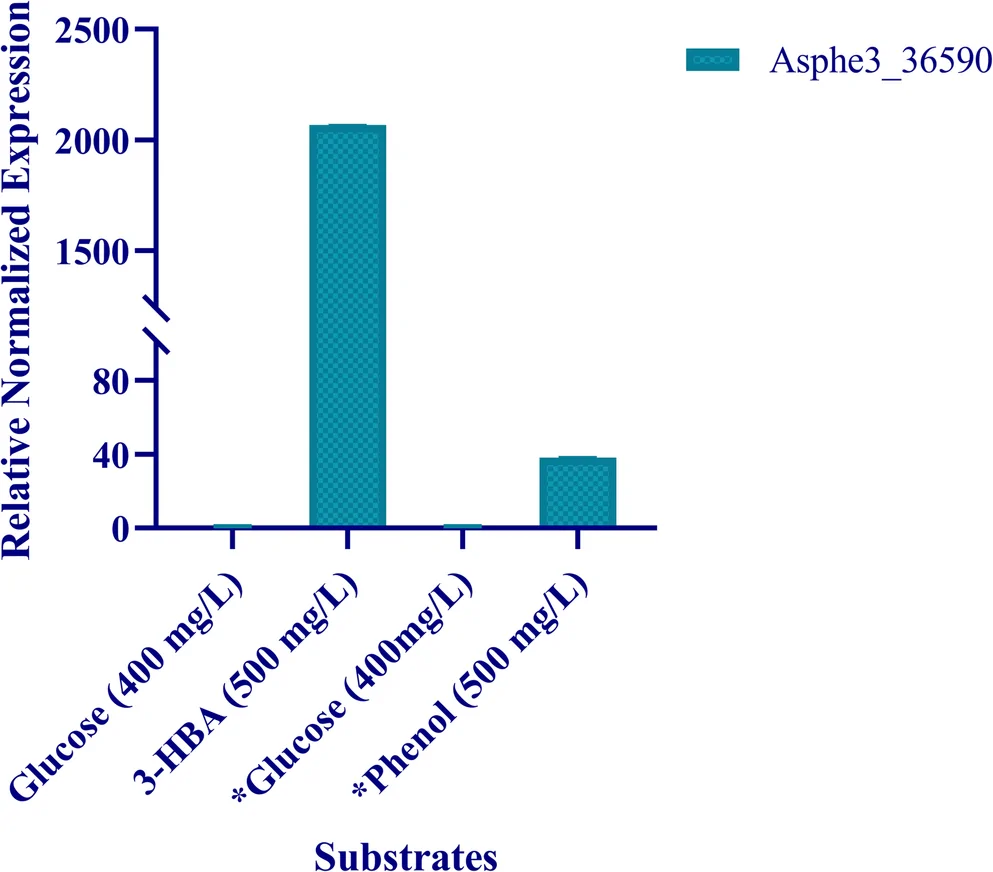
Relative normalized expression of Asphe3_36590 in glucose, 3-HBA and phenol substrates. Asterisks indicate data reported in previous study

The transcription levels of the gene were assessed by RT-qPCR when the strain was grown in 500 mg/L of 3-HBA. Specifically, Asphe3_36590 showed a striking transcriptional induction of about 2000 times when Sphe3 cells were grown in the presence of 3-HBA when compared to glucose (Fig. 3, Table S4). Instead, as previously reported, when Sphe3 cells were grown in the presence of phenol, the same gene showed a 38-fold change increase in mRNA expression levels when compared to glucose (Asimakoula et al. 2023).

### Biochemical properties of 3HB4H

To verify experimentally the above results, the 1905 bp Asphe3_36590 gene from Sphe3 was amplified by PCR, cloned, and expressed as a His-tagged protein in *E. coli* BL21 (DE3) using the pET29c(+) overexpression vector. The heterologous expression of the recombinant gene resulted in a protein band of ~ 69 kDa, as determined by sodium dodecyl sulfate-polyacrylamide gel electrophoresis (SDS-PAGE) (Figure S3), corresponding well to the predicted Mw of 69.37 kDa.

The addition of exogenous FAD to the purified fraction of the putative 3HB4H restores the activity of the purified enzyme, which appears to lose the flavonoid cofactor during its purification, and consequently its activity. Specifically, optimum enzyme activity was achieved after incubating the enzyme with 0.25 mM FAD. Loss of enzyme activity during purification has been reported previously for other flavin-dependent enzymes (Wang et al. 1987; Suarez et al. 1995; Park et al. 2007). The purified enzyme after its heterologous expression in *E. coli* BL21 (DE3) cells exhibited specific activity of 0.82 U·mg^− 1^ protein.

The recombinant 3HB4H from Sphe3 exhibited an optimum activity at pH 8.5 and 30 °C. In contrast, both 3HB4Hs from *Comamonas testosteroni* and *Chromohalobacter* sp. HS-2 (MobA) showed their highest activity at lower pH, 7.3 and 7, respectively (Chen et al. 1998; Kim et al. 2012). Kim et al. presented comparable data regarding the optimal temperature for the activity of 3HB4H from *Chromohalobacter* sp. HS-2 (MobA), which was found at 25 °C (Kim et al. 2012).

As presented in Table 1, all 3-HBA hydroxylases require FAD as a prosthetic group, while the Km values for the substrate range at similar levels. The purified Sphe3 3HB4H displayed Michaelis–Menten kinetics with an apparent Km 72.6 ± 10.4 µM and Vmax 42.5 ± 1.4 µM·min^− 1^ as estimated at 25 °C. The Km value of 3HB4H for 3-HBA is higher than the Km of 3HB4H from *C. testosteroni* (30 µΜ) and similar to the Km of 3HB4H from *Chromohalobacter* sp. HS-2 (80 µΜ), revealing a rather good affinity with 3-HBA as the substrate.

As far as the cofactor affinity is concerned, 3HB6Hs appear to prefer NADH, whereas 3HB4Hs can utilize NADPH and/or NADH as electron donor (Table 1). 3HB4H from Sphe3 appears to utilize both coenzymes NADH and NADPH, exhibiting similar Km and Vmax values for both. On the other hand, 3HB4H from *C*. *testosteroni* holds a higher affinity for NADPH (Km = 70 µM) compared to NADH (Km = 3000 µM) (Michalover and Ribbons 1973), whereas 3HB4H (MobA) from *Chromohalobacter* sp. HS-2 has been reported to utilize only NADH (Kim et al. 2012).

**Table 1:** Characteristics for 3-HBA hydroxylases from various strains

Enzyme	Strain	FAD	NADH/ NADPH	Km (µM) for 3-HBA	Km (µM) for NADH/NADPH	Reference
3HB4H (MobA)	*P. phenanthrenivorans Sphe3*	+	NADH/ NADPH	72.6	40.2 (NADH) / 40 (NADPH)	This paper
	*C. testosteroni*	+	NADPH	30	3000 (NADH) / 70 (NADPH)	(Michalover and Ribbons 1973; Hiromoto et al. 2006)
	*Chromohalobacter* sp. HS-2	+	NADH	80	-	(Kim et al. 2012)
	*A. niger* *	+	NADPH	190**	200**	(Premkumar et al. 1969)
3HB6H (PobA)	*Β. cepacia*	+	NADH/ NADPH	40	162 (NADH) / 168 (NADPH)	(Wang et al. 1987)
	*Micrococcus sp.*	+	NADH	63	95	(Rajasekharan et al. 1990)
	*K. pneumoniae M5a1*	+	NADH	-	-	(Suarez et al. 1995)
	*P. alcaligenes NCIMB 9867*	+	NADH	79	112 (NADH) / 225 (NADPH)	(Gao et al. 2005)
	*P. naphthalenivorans CJ2*	+	NADH	64.9	493	(Park et al. 2007)
	*C. glutamicum ATCC 13,032*	+	NADH	53.4	-	(Yang et al. 2010)
	*R. jostii RHA1*	+	NADH	46	48	(Montersino and Van Berkel 2012)
	*Martelella sp. AD-3*	+	NADH	104.1	72.6	(Chen et al. 2018)

* *Aspergillus niger*: fungus ** Partially purified enzyme-cell extract

Incubation with various concentrations of several divalent cations (Ag^+^, Ba^2+^, Ca^2+^, Fe^2+^, Mg^2+^, Mn^2+^, Zn^2+^) for 30 min exhibited inhibitory effects for Sphe3 3HB4H, leading to a total loss of enzymatic activity at all cases. Similarly, the activities of MobA from *Chromohalobacter* sp. HS-2 and 3HB6H from *K. pneumoniae M5a1* were significantly decreased after incubation with Fe^2+^ (Suarez et al. 1995; Kim et al. 2012). Nevertheless, the activity of Sphe3 3HB4H was enhanced after incubation for 30 min with 1 mM cobalt ions (Co^2+^) (133% relative activity), as also reported before for MobA from *Chromohalobacter* sp. HS-2 under the same conditions (Kim et al. 2012).

### NMR investigation of 3-HBA-catalyzed biotransformation across biological systems

#### In Situ NMR monitoring of 3-HBA hydroxylation by purified 3HB4H

**Fig. 4:**
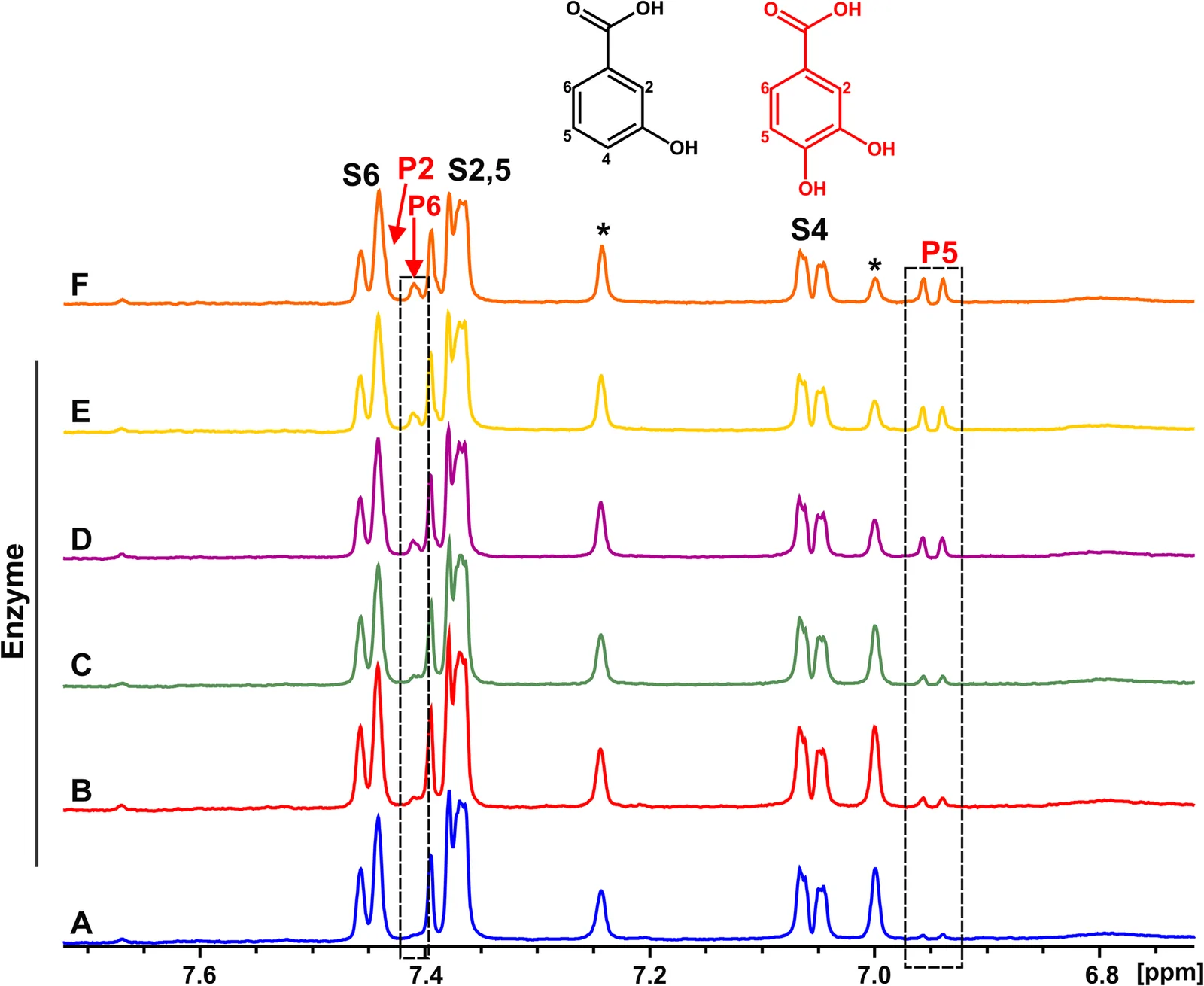
In situ monitoring of the biotransformation of the substrate 3-HBA (0.3 mM), denoted with S, to the product PCA, denoted with P, by 3HB4H (0.02 µM) in the NMR tube. (**A**) ^1^H NMR spectra 5 min, (**B**) 10 min, (**C**) 15 min, (**D**) 1.2 h (**E**) 1.5 h and (**F**) 2.5 h after the addition of 3-HBA to the enzyme reaction solution. Numbers following S and P correspond to the proton numbering shown in the molecular structures. Peaks belonging to NADH and imidazole are indicated by asterisks

The enzymatic conversion of 3-HBA by recombinant 3HB4H was investigated in situ using advanced NMR spectroscopy to monitor substrate turnover and product formation. 1D and 2D NMR spectra were recorded, identifying PCA as the reaction product. Figure 4 illustrates the temporal progression of the enzymatic conversion of 3-HBA by purified recombinant 3HB4H, monitored at specific intervals (5 min, 10 min, 15 min, 1.2 h, 1.5 h, and 2.5 h). The initial ^1^H NMR spectrum, recorded 5 min post-reaction initiation, exhibited distinct proton signals characteristic of the substrate 3-HBA, annotated in black. Concurrently, two emerging doublets at 6.95 and 7.42 ppm, were assigned to the aromatic protons H5 and H6 of PCA, respectively (Fig. 4). Additionally, a shoulder peak at 7.44 ppm, corresponding to proton H2 of PCA became more pronounced after 1 hour of reaction. Substrate conversion to PCA reached a plateau at 2.5 h and a stable product-to-substrate integral ratio (PCA H5/3-HBA H4) of approximately 0.3 was observed, indicating partial conversion of 3-HBA under the in vitro conditions (Fig. 4F).

To fully assign the PCA reaction product, 2D ^1^Η-^1^Η TOCSY, ^1^Η-^13^C HSQC and ^1^Η-^13^C HMBC NMR spectra were acquired. The ^1^H-^1^H TOCSY NMR spectrum (Figure S4), revealed a characteristic cross-correlation peak between protons H5 and H6 of PCA. 2D ^1^-H^13^ C HSQC and HMBC NMR spectra (Figure S5) facilitated the complete assignment of the carbon chemical shifts for PCA, which were in accordance with our previous work (Tsagogiannis et al. 2021) and independent reference NMR spectral data reported for PCA (HMDB ID HMDB0001856, Wishart et al. 2022; BMRB, entry bmse000328). The chemical shifts at 128.7 ppm, 117.22 ppm, 143.4 ppm, 147.6 ppm, 115.3 ppm and 122.16 ppm were attributed to carbons C1, C2, C3, C4, C5 and C6, respectively.

#### NMR monitoring of 3-HBA hydroxylation in *E. coli* cell crude extract

**Fig. 5:**
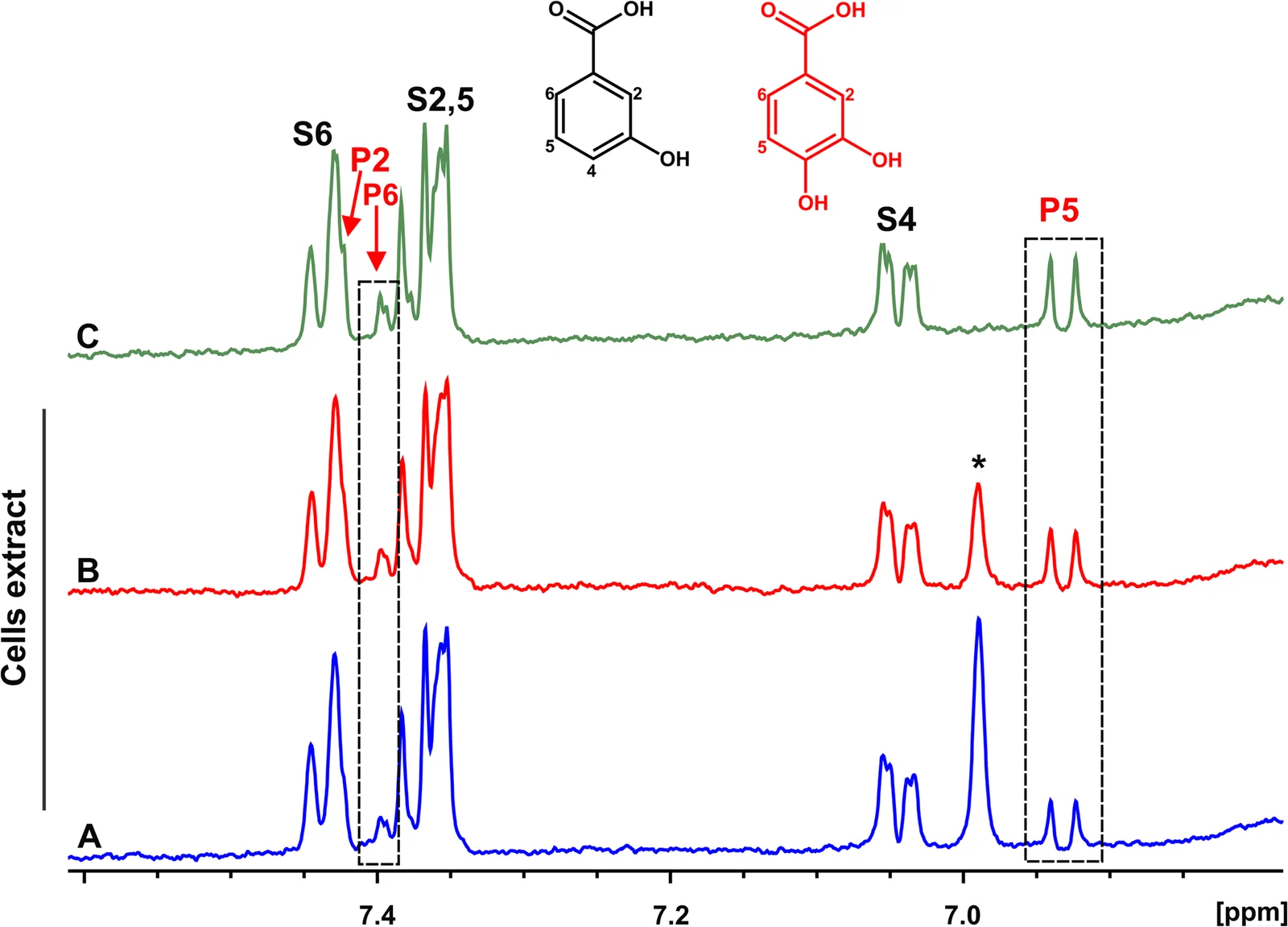
^1^H NMR spectra of the biotransformation reaction of the substrate 3-HBA (0.3 mM), denoted with S, to the product PCA, denoted with P, by 3HB4H within cell extract system (**A**) 5 min, (**B**) 10 min and (**C**) 1 h after the reaction initiation. Numbers following S and P correspond to the proton numbering shown in the molecular structures. Peaks belonging to NADH are indicated by asterisk

To better simulate the physiological conditions, the enzymatic biotransformation of 3-HBA was investigated in situ within a cell crude extract system. ^1^H NMR spectra were of excellent resolution and both substrate and hydroxylated product were clearly assigned. This approach underscores the utility of cell extract systems in preserving native enzymatic conditions and enhancing the physiological relevance of biotransformation studies. A plateau was reached 1 h after the reaction initiation and a stable product-to-substrate integral ratio (PCA H5/3-HBA H4) of approximately 1 was obtained, indicating partial conversion of 3-HBA under these conditions (Fig. 5C).

#### In-cell NMR investigation of intracellular 3-HBA biotransformation by 3HB4H

**Fig. 6:**
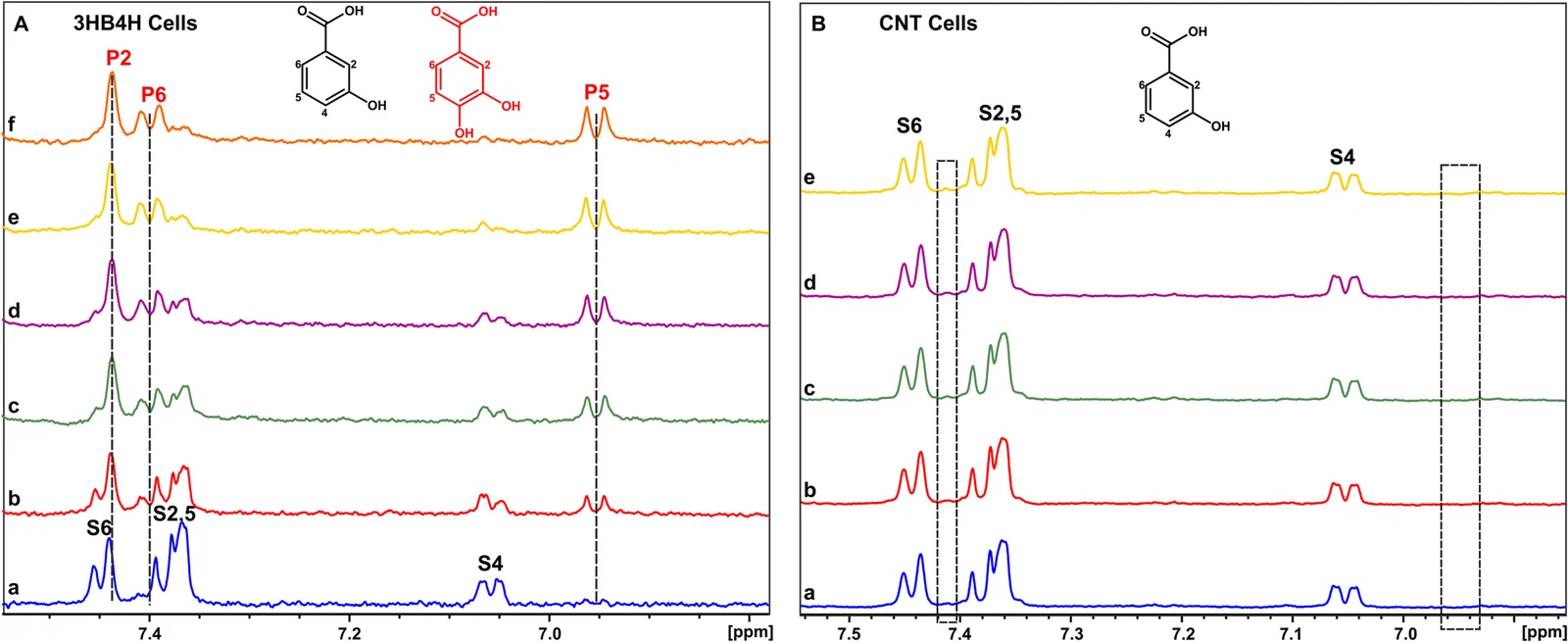
(**A**) In-cell NMR monitoring of the substrate 3-HBA (0.2 mM), denoted with S, hydroxylation to the product PCA, denoted with P by 3HB4H within living *E. coli* cells overexpressing the enzyme (A) 5 min (a), 10 min (b), 15 min (c), 25 min (d), 35 min (e) and 1 h (f) after the reaction initiation. (**B**) ^1^H NMR spectra of 3-HBA (0.3 mM) in *E. coli* cells without the 3HB4H plasmid 5 min (a), 10 min (b), 20 min (c), 25 min (d) and 30 min (e) after the addition of the substrate. Numbers following S and P correspond to the proton numbering shown in the molecular structures

To gain a better understanding of the physiological enzymatic context, circumvent labor-intensive experimental steps, and provide insights into the dynamics of hydroxylation under physiologically relevant conditions, we evaluated the feasibility of real-time reaction monitoring within an intracellular environment. The hydroxylation reaction was initiated by adding 0.2 mM of 3-HBA substrate to the solution of living cells suspension, and ^1^H NMR spectra were recorded in real-time within the NMR tube (Fig. 6A). Remarkably, high-resolution spectra were achieved, enabling unambiguous identification of both substrate and product signals and the clear observation of the hydroxylation progress without the interference from cellular background signals in the aromatic ppm region. Signals corresponding to protons H5 and H6 of PCA were detected as early as 5 min after the reaction initiation and the reaction proceeded to near-complete conversion within 1 hour, corresponding to > 95% apparent conversion based on NMR integral ratios (PCA H5/3-HBA H4) (Fig. 6Α).

**Fig. 7:**
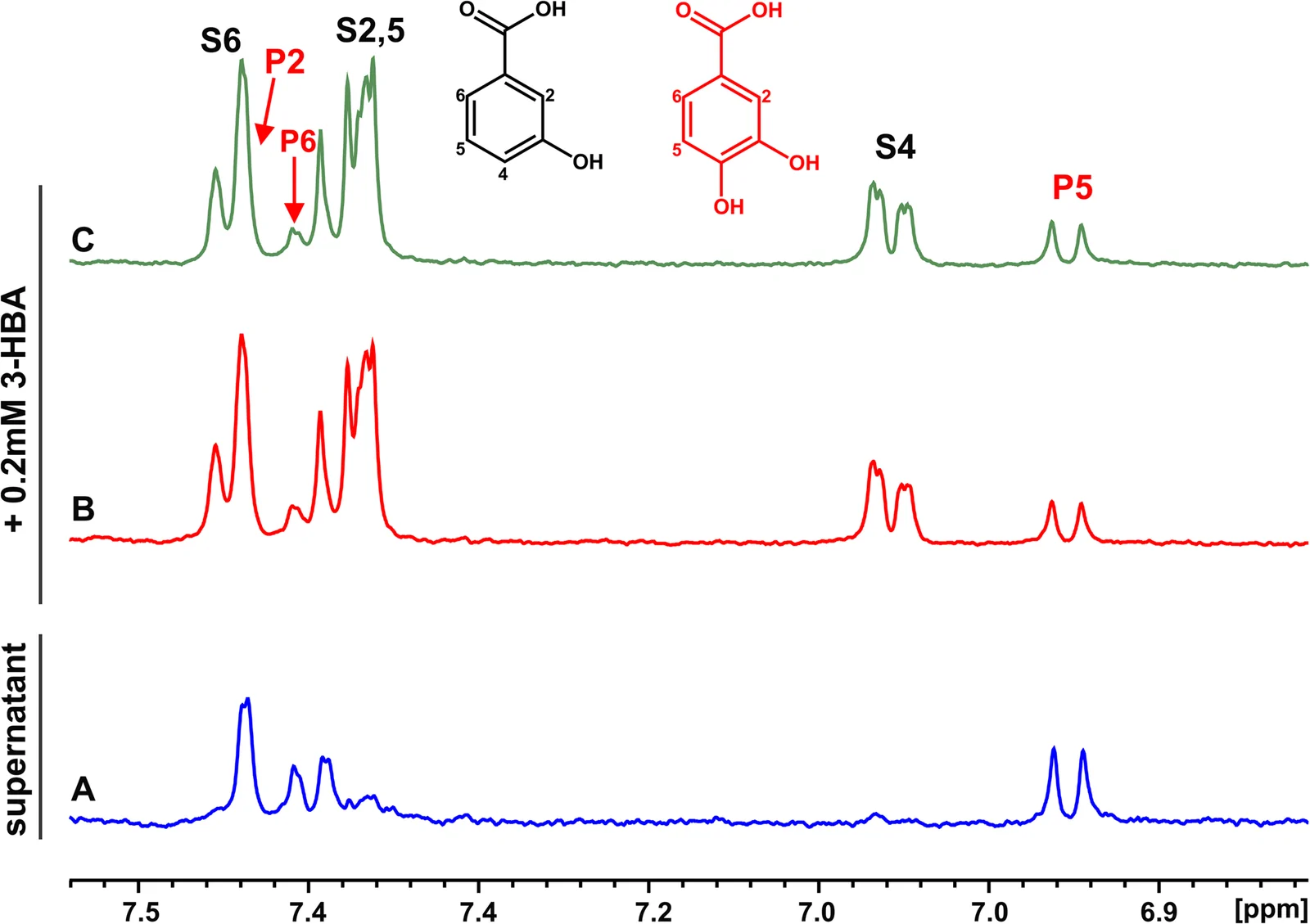
^1^H NMR spectrum of the supernatant of *E. coli* cells overexpressing 3HB4H after treatment with 0.2 mM of substrate 3-HBA for 2 h in the NMR spectrometer (**A**). ^1^H NMR spectra of sample A 5 min (**B**) and 45 min (**C**) after the addition of fresh solution of 0.2 mM 3-HBA. The substrate 3-HBA is denoted with S and the product PCA with P. Numbers following S and P correspond to the proton numbering shown in the molecular structures

To validate the stability of 3-HBA inside living bacterial cells lacking 3HB4H enzyme, control experiments were conducted using *E. coli* BL21-pET29c cells without the 3HB4H insert. Cell densities were optimized independently for each in-cell NMR condition to ensure adequate spectral quality and cell integrity. A lower OD₆₀₀ was used for 3HB4H-expressing cells to minimize line broadening and signal overlap associated with recombinant protein overexpression at higher cell densities, whereas control cells lacking recombinant protein expression could be analyzed at higher OD₆₀₀ to improve signal detectability without compromising spectral resolution. Subsequently, ^1^H NMR spectra revealed no changes in substrate levels, confirming that hydroxylation of 3-HBA to PCA occurs exclusively in the presence of 3HB4H enzyme (Fig. 7B). To further substantiate that the enzymatic biotransformation of 3-HBA was confined to the intracellular compartment and did not occur in the extracellular medium, *E. coli* cells overexpressing 3HB4H enzyme which were treated with 0.2 mM of substrate, were subsequently spun down and separated from the supernatant. Fresh solution of 3-HBA was subsequently added into the supernatant solution. No hydroxylation activity was detected in the supernatant 5 min (Fig. 7B) and 45 min (Fig. 7C) after the addition, indicating that catalytic activity was confined to intact cells and that biotransformation occurred intracellularly rather than in the surrounding medium (Fig. 7). Together with the observed substrate conversion exclusively in 3HB4H-expressing cells, these findings support that 3-HBA was sufficiently accessible to the intracellular enzyme under the assay conditions. Furthermore, a cellular viability assay was conducted before and after NMR analysis. Plating of *E. coli* cells verified that they remained viable after the completion of the NMR experiments, ensuring the physiological relevance of the observations (Figure S6).

These findings not only confirm the successful enzymatic biotransformation of 3-HBA to PCA intracellularly but also highlight the utility of ^1^H NMR spectroscopy in monitoring enzymatic hydroxylation reactions directly within living bacterial cells. This approach eliminates the need for time-consuming cell lysis and enzyme purification steps, allowing for the preservation of native enzymatic conditions. High-resolution NMR spectra enabled the unambiguous detection of both substrate and product, confirming the efficiency of 3HB4H enzyme in catalyzing the conversion of 3-HBA to PCA intracellularly. The absence of hydroxylation in control cells lacking the enzyme further validated the specificity of the reaction. The current study provides a robust framework for studying enzymatic biotransformation in living cells systems, offering insights into metabolic processes under physiologically relevant conditions.

Collectively, in-cell NMR spectroscopy revealed a substantially higher extend of conversion as indicated by product-to-substrate integral ratio (PCA H5/3-HBA H4 ≈ 10.7) within 1 h compared to the ratios observed for the purified enzyme (≈ 0.3 after 2.5 h) and crude cell extracts (≈ 1.0 after 1 h). This enhanced intracellular turnover could be attributed to the higher effective concentration of the overexpressed 3HB4H enzyme, the continuous regeneration of essential cofactors within the living cells, and the absence of inhibitory components that may be present in cells extract or during enzyme purification. These findings underscore the unique value of in-cell NMR in capturing enzymatic efficiency under physiological conditions.

#### NMR screening of alternative substrates recognition by 3HB4H

To determine the substrate preference of the enzyme 3HB4H, its catalytic activities were tested with a range of different substrates by observing spectral changes of enzymatic reactions with UV/Vis spectroscopy, whereas in-cell NMR spectroscopy was used for the confirmation of substrate recognition and enzymatic turnover. Cell free crude extracts with the overexpressed 3HB4H were used in reaction with potential substrates 4-ΗΒΑ, 2-ΗΒΑ, gentisic acid, phthalic acid, PCA and phenol, as described in Materials and Methods, and spectral changes of each reaction in UV/Vis were recorded. No changes were observed for 2-ΗΒΑ, phthalic acid, PCA and phenol, while 3HB4H seemed to recognize 4-HBA and gentisate apart from 3-HBA. Purified 3HB4H exhibited 0.08 and 0.02 U·ml^− 1^ of activity, by measuring NADH oxidation at 340 nm, in the presence of 4-HBA and gentisate, respectively. Similarly, 3HB4H from *C. testosteroni* has been reported to utilize several dihydroxybenzoates, one of them being gentisate (or 2,5-dihydroxybenzoic acid) resulting in 2, 4, 5-trihydrobenzoate as reaction product (Michalover and Ribbons 1973). On the contrary, MobA from *Chromohalobacter* sp. HS-2 showed specific activity only with 3-HBA (Kim et al. 2012).

**Fig. 8:**
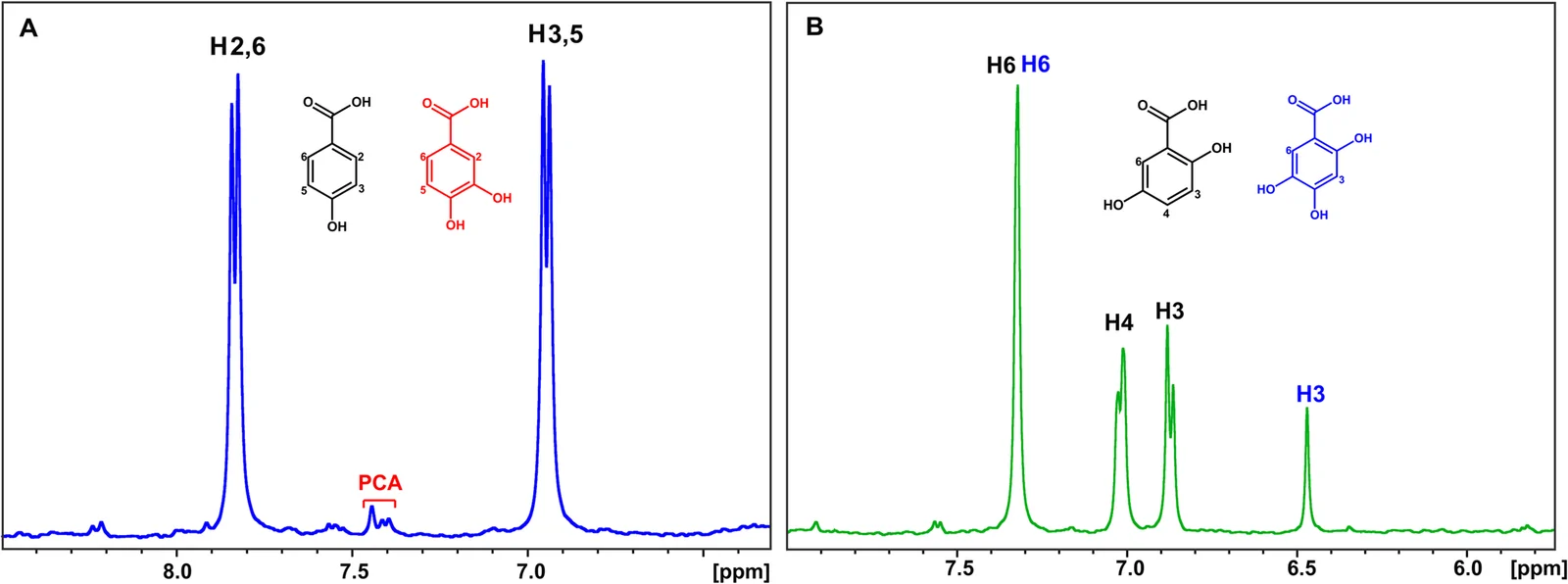
In-cell ^1^H NMR spectra of the biotransformation reaction of 4-HBA (black color) to PCA (red color) (**A**) and of gentisic acid (black color) to 2,4,5-trihydroxybenzoic acid (blue color) (**B**) by 3HB4H enzyme in living *E. coli* cells

The above results were verified by in-cell NMR spectroscopy within living *E. coli* cells overexpressing the enzyme 3HB4H. The catabolism of 4-HBA yielded PCA at a relatively low concentration (Fig. 8A), whereas gentisic acid was converted to 2,4,5-trihydroxybenzoic acid with proton H3 appearing at 6.5 ppm and proton H6 overlapping at 7.3 ppm with proton H6 of gentisic acid (Fig. 8B). Notably, the enzyme did not recognize other substrates such as phenol, as no product formation was detected in their ^1^H NMR spectra within intact bacterial cells.

The product analysis by in-cell NMR spectroscopy confirmed that Asphe3_36590 gene encodes for 3HB4H and not for a phenol monooxygenase, even though their amino acid sequences share high identity as it has been reported before (Asimakoula et al. 2023). These findings are consistent with previously reported substrate specificity of 3HB4H, further substantiating its enzymatic versatility. MobA from *Comamonas testosteroni* GZ39 converts *m*-hydroxybenzoate and to a lesser extent *p*-hydroxybenzoate to protocatechuate, whereas it is known that MobA lacks the ability to transform phenol (Chang and Zylstra 2008). Therefore, phenol was also examined by both UV/Vis and in-cell NMR and the results were consistent with the literature, since no interaction or biotransformation was detected (Figure S7).

## Conclusions

The combination of structure prediction analysis, assessment of the transcriptional levels of the gene and functional characterization of the enzyme, led to the conclusion that the gene with the locus tag Asphe3_36590 actually encodes a 3HB4H enzyme that even though it shares a high identity with domains often found in phenol monooxygenases, it does not identify phenol as substrate.

To the best of our knowledge, this is the first report on biochemical and kinetic characterization of a 3-hydroxybenzoate 4-hydroxylase in the genus *Pseudarthrobacter*. 3HB4H from *P*. *phenanthrenivorans* Sphe3 was overexpressed, purified, and characterized for its biochemical and kinetic properties.

In situ NMR monitoring confirmed the enzymatic hydroxylation of 3-HBA to PCA, enabling precise identification and assignment of the reaction product signals. Time-resolved NMR experiments performed with purified enzyme, crude cell extracts, and living recombinant cells revealed distinct product-to-substrate integral ratios, reflecting different extents of biotransformation across the three experimental systems. While only partial substrate conversion was achieved with purified enzyme and cell extracts, in-cell NMR experiments demonstrated nearly complete substrate conversion within one hour under near-physiological intracellular conditions. These differences in conversion across purified enzyme, cell extracts, and whole-cell systems highlight the influence of the biological context on 3HB4H activity and provide complementary functional insights under progressively more native-like conditions. The enhanced catalytic efficiency observed in intact cells likely reflects the favorable intracellular environment, including endogenous cofactor regeneration, improved cofactor availability, and preservation of enzyme stability and functionality.

These observations further highlight the utility of the in-cell NMR approach for monitoring enzymatic biotransformations under physiologically relevant intracellular conditions, where native cofactor regeneration, molecular crowding, and cellular homeostasis can better preserve catalytic activity compared with purified in vitro systems.

High-resolution in-cell NMR spectroscopy enables direct and non-invasive observation of complex biochemical processes with high specificity, while preserving cellular integrity and metabolic activity. This methodology constitutes a pivotal advancement in enzymology by enabling rapid and cost-efficient characterization of biocatalytic processes, elucidating metabolic pathways, and supporting the optimization of enzymatic functions for biotechnological applications.

## Supplementary Information

Below is the link to the electronic supplementary material.

Supplementary Material 1 (DOCX 1.50 MB)

## Data Availability

All experimental data pertinent to this manuscript is accessible for review, either in the manuscript, in a public database, or as material uploaded with the manuscript as additional files for review purposes.
