# Supplementary material for: Revealing 3-hydroxybenzoate-4-hydroxylase from Pseudarthrobacter phenanthrenivorans Sphe3 via In-Cell NMR analysis and molecular cloning
Source: Int Microbiol. 2026 Jun 26;29(7):1379–95. doi: 10.1007/s10123-026-00856-7 (PMC13653839; doi:10.1007/s10123-026-00856-7)
Supplement: Supplementary file 1 [file 10123_2026_856_MOESM1_ESM.docx]

**Supporting information**

**Table S1.** Oligonucleotides used for the RT-qPCR experiments.

| **Gene Locus Tag/**  **Gene Name** | **Oligonucleotides Designation** | **Oligonucleotides Sequence (5’→3’)** | **Size (bp)** |
| --- | --- | --- | --- |
| Asphe3_36590 | phehydrox_for_ | GTCACCGACTTCCCCGATAT | 167 |
|  | phehydrox_rev_ | ATCTGCTCGATGGTGGTGTT |  |
| Asphe3_00060/*gyr*β | gyrβ_for_ | GGCTAACGACAATACAGATA | 210 |
|  | gyrβ_rev_ | ACCACTTCATAAACAAGGT |  |

**Table S2.** BlastP alignment results of Sphe3 3HB4H and other reported flavoprotein monooxygenases.

| **Description** | **Max Score** | **Total Score** | **Query Cover** | **E value** | **Per. Ident** | **Acc. Len** | **Accession** |
| --- | --- | --- | --- | --- | --- | --- | --- |
| 3HB4H [*Comamonas* *testosteroni* GZ39] | 625 | 625 | 98% | 0.0 | 50.62% | 639 | AAR25885.1 |
| 3HB4H [*Chromohalobacter* sp. HS-2] | 622 | 622 | 99% | 0.0 | 49.39% | 651 | AFK24467.1 |
| 3HB4H [*Comamonas* *testosteroni* KH122-3S] | 622 | 622 | 98% | 0.0 | 50.70% | 639 | BAF34928.1 |
| 3HB4H [*Comamonas* *testosteroni* ATCC 17454] | 618 | 618 | 98% | 0.0 | 50.16% | 639 | ABN58516.1 |
| Phenol hydroxylase [*Cutaneotrichosporon* *cutaneum*]* | 355 | 355 | 93% | 7e-117 | 35.71% | 665 | AAA34202.1 |
| Phenol 2-monooxygenase [*Pseudomonas* sp. EST1001] | 81.3 | 81.3 | 58% | 5e-19 | 26.99% | 607 | P31020.1 |
| Phenol 2-monooxygenase [*Pseudomonas* *pickettii* PKO1] | 48.1 | 80.9 | 17% | 2e-08 | 40.26% | 671 | Q01551.2 |
| 4HB3H [*Sinomonas* *cyclohexanicum*] | 37.7 | 37.7 | 36% | 2e-05 | 24.79% | 396 | BAE46572.1 |
| 3HB6H [*Aquipseudomonas* *alcaligenes*] | 37.0 | 37.0 | 31% | 3e-05 | 25.00% | 394 | Q9F131.1 |
| SalA [*Stutzerimonas* *stutzeri*] | 34.3 | 34.3 | 27% | 2e-04 | 28.88% | 389 | AAD02157.1 |
| 3HB6H [*Polaromonas* *naphthalenivorans* CJ2] | 33.9 | 33.9 | 49% | 3e-04 | 21.81% | 400 | Q3S4B7.1 |
| 4HB3H [*Rhodococcus* *rhodnii*] | 30.8 | 30.8 | 14% | 0.003 | 25.81% | 396 | AHF21003.1 |
| 4HB3H [*Rhodococcus* *opacus*] | 30.4 | 30.4 | 12% | 0.004 | 25.97% | 392 | ANS30736.1 |
| 4HB3H [*Pseudomonas* *putida*] | 30.0 | 30.0 | 10% | 0.005 | 28.99% | 395 | CAB64666.1 |
| SalA [*Sphingomonas* sp.] | 29.3 | 29.3 | 8% | 0.008 | 35.09% | 395 | BAA19150.1 |
| 3HB6H [*Martelella* sp. AD-3] | 29.3 | 29.3 | 58% | 0.009 | 24.14% | 403 | AMM83381.1 |
| 4HB3H [*Rhodococcus* *opacus*] | 28.9 | 28.9 | 14% | 0.011 | 23.66% | 392 | AHF21002.1 |
| SalA [*Acinetobacter* *baylyi* ADP1] | 27.3 | 27.3 | 27% | 0.032 | 22.86% | 423 | AAF04312.1 |
| 3HB6H [*Klebsiella* *pneumoniae*] | 27.3 | 27.3 | 26% | 0.035 | 22.91% | 397 | AAW63416.1 |
| SalA [*Pseudomonas* *putida* ND6] | 26.9 | 26.9 | 8% | 0.049 | 30.36% | 434 | AAP44222.1 |

**Yeast strain*.

**Figure S1.** InterProScan functional analysis of Asphe3_36590 protein. Entries are presented with color by accession. Families’ entries: phenol monooxygenase (blue), rifampicin-like monooxygenase (green) and aromatic-ring hydroxylase (flavoprotein monooxygenase) signatures (light blue). Domains’ entries: FAD/NAD(P)-binding domain superfamily (pink), FAD-binding domain (purple), phenol hydroxylase-like (C-terminal dimerization domain) (light green), thioredoxin-like superfamily (red) and phenol hydroxylase (C-terminal TRX-fold domain superfamily) (dark blue).

**Table S3.** InterProScan functional analysis of Asphe3_36590 protein.

|  | **Accession** | **Short Name** | **Name** | **Matches (aa positions)** | **Source database** |
| --- | --- | --- | --- | --- | --- |
| **Representative families** | | | | | |
|  | NF006144 | PRK08294.1 | phenol 2-monooxygenase | 1-624 | NCBIFAM entry |
|  | IPR050641 | RIFMO-like | Rifampicin monooxygenase-like | 32-605 | InterPro |
|  | PR00420 | RNGMNOXGNASE | aromatic-ring hydroxylase (flavoprotein monooxygenase) signature | 34-56, 197-212, 337-352, 352-368, 370-388, 388-404 | PRINTS entry |
| **Representative domains** | | | | | |
|  | IPR002938 | FAD-bd | FAD-binding domain | 32-402 | InterPro |
|  | IPR036188 | FAD/NAD-bd_sf | FAD/NAD(P)-binding domain superfamily | 33-218, 314-417; 34-101, 318-400, 132-222 | InterPro |
|  | IPR036249 | Thioredoxin-like_sf | Thioredoxin-like superfamily | 443-627 | InterPro |
|  | IPR012941 | Phe_hydrox_C_dim_dom | Phenol hydroxylase-like, C-terminal dimerisation domain | 439-625 | InterPro |
|  | IPR038220 | PHOX_C_sf | Phenol hydroxylase, C-terminal TRX-fold domain superfamily | 419-624 | InterPro |

**Figure S2.** Ramachandran plot validation of the tertiary structure model. The backbone stereochemical quality was evaluated using MolProbity, showing a distribution of dihedral torsion angles (ϕ and ψ) across four distinct residue categories: **(A)** General residues, **(B)** Glycine-specific regions, **(C)** Proline-specific regions, and **(D)** Pre-Proline residues. Fully shaded areas represent the favored regions (94.53% overall), while contour lines define the additionally allowed boundaries. A highly minimal outlier rate of 0.80% confirms the physical and geometric reliability of the modeled backbone.

**Table S4.** Relative normalized expression of Asphe3_36590.

| **Substrates** | **Relative Normalized Expression of Asphe3_36590** | **SD** | **N** |
| --- | --- | --- | --- |
| Glucose (400 mg/L) | 1 | 0,5 | 3 |
| 3-HBA (500 mg/L) | 2066,6 | 0,2 | 3 |
| *Glucose (400mg/L) | 1 | 0,4 | 3 |
| *Phenol (500 mg/L) | 38,1 | 0,4 | 3 |

* Data acquired from previous study.

**Figure S3**. SDS-PAGE protein analysis. Purification of overexpressed 3HB4H from *P. phenanthrenivorans* Sphe3. Lane M, Unstained Protein Standard, Broad Range (10–200 kDa) 10–20% Tris-glycine (ΝΕΒ); lane 1, cell-free crude extract from *E. coli* BL21/pET29c::Asphe3_36590; lane 2, column chromatography flow-through; lanes 3-9, purified 3HB4H protein (approx. 69 kDa) fractions eluted with varying concentrations of imidazole (100-250 mM).

**Figure S4.** ^1^Η-^1^Η TOCSY 2D NMR of the biotransformation reaction of 3-HBA (0.3 mM) to PCA by 3HB4H.

**Figure S5.** ^1^Η-^13^C HSQC 2D NMR of the biotransformation reaction of 3-HBA (0.3 mM) to PCA by 3HB4H. Peaks belonging to NADH and imidazole are indicated by asterisks.

**Figure S6.** **Νutrient agar plates showing bacterial cell viability before and after NMR spectroscopy.** Serial dilutions were performed in triplicate, with a precise drop volume of 50 μl plated for each dilution.

**Figure S7.** In-cell ^1^H NMR spectrum of phenol in the presence of 3HB4H-expressing *E. coli* cells. No evidence of phenol biotransformation was observed under the experimental conditions.
